# Weight Cycling Deregulates Eating Behavior in Mice via the Induction of Durable Gut Dysbiosis

**DOI:** 10.1002/advs.202501214

**Published:** 2025-06-26

**Authors:** Mélanie Fouesnard, Adélie Salin, Sandy Ribes, Magali Monnoye, Gaëlle Champeil‐Potokar, Marie‐Sabelle Hjeij, Gwénaëlle Randuineau, Léa Le Gleau, Selma Ben Fradj, Catherine Philippe, Alexandre Benani, Isabelle Denis, Véronique Douard, Gaëlle Boudry

**Affiliations:** ^1^ Institut Numecan INRAE INSERM Univ Rennes Rennes France; ^2^ Institut MICALIS INRAE AgroParisTech Université Paris‐Saclay Jouy‐en‐Josas France; ^3^ UMR PNCA INRAE AgroParisTech Université Paris‐Saclay Palaiseau France; ^4^ Centre des Sciences du Goût et de l'Alimentation UMR CNRS U6265 INRAE U13241 Université de Bourgogne Dijon France

**Keywords:** eating behavior, hedonic appetite, microbiota‐gut‐brain axis

## Abstract

Background & Aims: Alternating periods of excessive and restrained eating results in weight cycling (‘yo‐yo’ effect), a suspected risk factor for eating behavior dysregulation such as binge eating. The hypothesis that recurrent diet alternation alters hedonic feeding regulation by changing either or both intestinal microbiota and brain neuronal and glial regulation in mouse is tested. **Methods**: C57BL/6 mice undergo 3 cycles of 1 week of western diet (WD) separated by 2 weeks of chow diet (CYCL group) or remain under chow diet (CTRL group). **Results**: CYCL mice exhibit weight cycling, with enhanced weight gain upon each WD feeding phase and increased energy intake specifically during the first hours following WD re‐introduction, reminiscent of binge‐eating episodes. Expression of reward‐related genes in the striatum and thickness of the astro‐glial barrier in the brain stem is enhanced in CYCL mice. Diet alternation induces caecal dysbiosis in CYCL mice. Gut microbiota transfer from CYCL mice to naive recipient mice recapitulates the altered eating behavior upon WD exposure. **Conclusions**: Alternation between high‐energy and standard diets is established to durably remodel the gut microbiota and the brain toward a profile associated with an increase in hedonic appetite and that this microbiota signature affects hedonic feeding regulation.

## Graphical abstract

Weight cycling in Humans is suspected to dysregulate eating behavior. Mice submitted to body weight cycling by alternating western‐type and regular chow diets display increased hedonic appetite when exposed to a palatable diet, reminiscent of binge‐eating episodes. Gut microbiota transfer from these mice to naive recipient ones recapitulates the altered eating behavior suggesting a role of the microbiota in weight regain after repeated dieting.

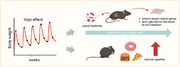

## 1 Introduction

The rise in overweight and obesity together with the social pressure for thinness increase the prevalence of dieting. Restrictive dieting practices have risen over the last few decades in Western countries and a recent meta‐analysis reported that 42% of adults from general population have attempted to lose weight.^[^ ^1^ ^]^ Moreover, first line obesity management relies on behavioral and dietary measures, including calorie‐restrictive regimens.^[^ ^2^ ^]^ However, long‐term studies report that restrictive dieting is ineffective in maintaining long‐term weight reduction and is susceptible to the ‘yo‐yo’ effect in which individuals experience weight cycling characterized by repeated episodes of weight loss and subsequent excessive weight regain.^[^ ^3^ ^]^ On average, individuals that lost weight through dietary intervention with or without exercise regained one‐third to half of the weight they lost within the first year after treatment and they will return to their baseline weight within 3–5 years after treatment.^[^ ^4^ ^]^ Surgical and pharmacological approaches also fail to sustain weight loss and result in the yo‐yo effect.^[^ ^5^, ^6^ ^]^ Weight cycling probably reflects physiological adaptations that would originally contribute to ensure increased survival capacity during repeated periods of feast and famine. However, in our modern societies, such adaptations can lead to adverse health consequences.^[^ ^7^ ^]^ More particularly, intense weight loss is accompanied by an increased orexigenic drive.^[^ ^8^ ^]^ Several clinical studies suggest that repeated dieting and weight cycling predispose to disordered eating, including binge eating.^[^ ^9^ ^]^ Hence, the greater the number of weight loss attempts, the greater the occurrence of binge eating.^[^ ^10^, ^11^ ^]^ However, these studies were cross‐sectional or retrospective, preventing any conclusion regarding the causal relationship between dieting and dysregulation of eating behavior. Therefore, preclinical models of binge eating and weight cycling are instrumental for studying such associations. While short‐time access (1 or 2‐hours per day) to a hypercaloric palatable diet induces an escalation of palatable diet consumption in rodents, reminiscent of binge eating, this model does not recapitulate weight cycling. In contrast, alternating extended periods (days to weeks) of a hypercaloric and standard diets is associated with weight cycling, along with metabolic and behavioral adaptations.^[^ ^12^, ^13^ ^]^ Interestingly, these studies have underscored the key role of gut microbiota in post‐dieting weight regain in obesity, attributing this effect to changes in energy expenditure.

The gut microbiota plays a crucial role in energy metabolism, notably by fermenting dietary fiber into short‐chain fatty acids (SCFAs), and modulating inflammation.^[^ ^14^ ^]^ Since it profoundly impacts many physiological systems and behaviors,^[^ ^15^ ^]^ including eating behaviors^[^ ^16^, ^17^, ^18^, ^19^ ^]^ we sought to investigate whether weight cycling was associated with deregulation of food intake driven by a peculiar gut microbiota signature. Noteworthy, we investigated the impact of weight cycling before the installation of obesity to avoid the confounding effects of significant obesity that is associated with profound modifications of energy expenditure and metabolic alterations such as hyperglycemia, hyper‐leptinemia, or insulin resistance, all affecting food intake regulation. Hence, we exposed mice to three successive dietary switches between a standard rodent diet and a western diet (WD), monitoring food intake daily throughout the procedure. Hedonic appetite was previously demonstrated as clearly limited to the normal rest phase.^[^ ^20^ ^]^ Therefore, we specifically focused on palatable food intake after the onset of the light period, which we defined as hedonic appetite—referring to food intake driven by pleasure in the absence of energy deficit or physical hunger. To assess the consequences of weight cycling on the microbiota‐brain axis, brain, and microbiota analysis were performed after the three dietary switches. To causally link gut microbiota signatures to changes in eating behavior, we performed microbiota transfer into naive mice and determined the impact on eating behavior.

## 2 Results

### 2.1 Weight Cycling Leads to Transient Heightened Hedonic Appetite

**Figure 1:**
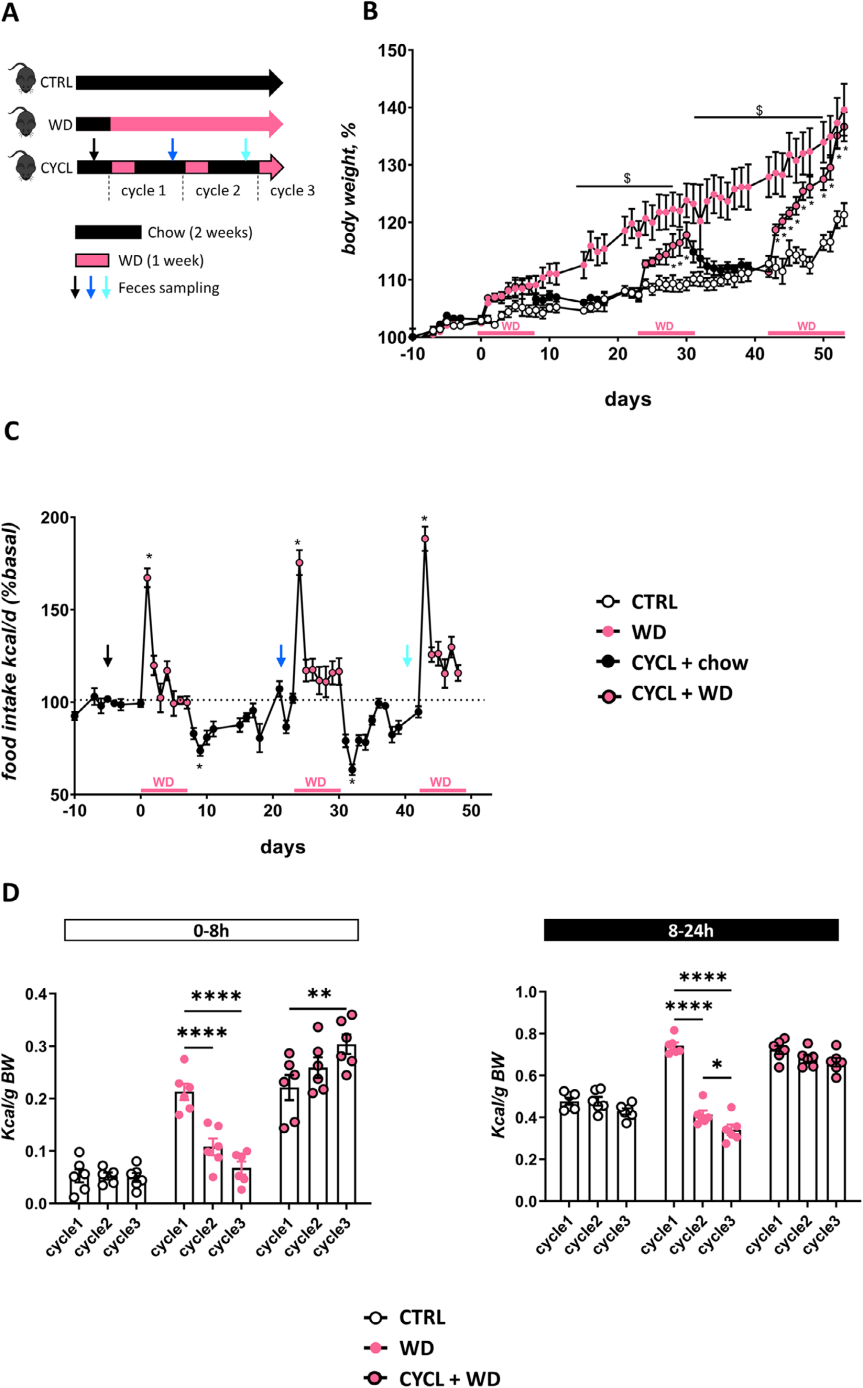
CYCL mice have altered regulation of energy intake upon WD introduction and display fecal dysbiosis. A) Experimental design: Mice were submitted to cycles of one week of western diet (WD) followed by 2 weeks of chow diet (CYCL mice) or left under chow diet (CTRL) or WD (WD). WD was introduced 2 hours after the onset of the light period. (B) Change in body weight of CTRL, WD, and CYCL mice during the whole protocol. C) Daily caloric intake of CYCL mice during the whole protocol expressed as a percentage of Kcal/day, relative to the average intake during the X days preceding the WD introduction. D) Caloric intake during the first 8 h (left panel) and the following 16 hours (right panel) of CTRL, WD, and CYCL mice, measured at the time of WD introduction for each cycle in the CYCL group. For figure 1B, *n* = 6 mice/group, *p < 0.05 between CTRL and CYCL mice and $*p* < 0.05 between WD and CYCL mice (Two‐way ANOVA followed by Tukey post‐hoc test). For figure 1B, *n* = 6 mice/group, *p < 0.05 compared to baseline (Mann‐Whitney test). For figures 1D, *n* = 6 mice / group, *p < 0.05, **p < 0.01, and ****p < 0.0001 (Two‐way ANOVA followed by Tukey post‐hoc test to compare food intake between cycles for each group). In CYCL mice, pink solid dots depict data when mice were under WD. Bars are means ± SEM.

To examine the impact of weight cycling on eating behavior, mice (referred to as CYCL mice) underwent 3 cycles of 1 week of WD each followed by 2 weeks of chow diet feeding (**Figure** **1**A), except on cycle 3 where they were left on WD to evaluate their kinetic of weight rebound. In response to WD, CYCL mice gained significantly more body weight in one week than CTRL (maintained on chow diet throughout the experiment) and WD mice (maintained on Western Diet throughout the experiment) during cycles 2 and 3 (Figure 1B; Figure S1, Supporting Information). CYCL mice lost weight each time they switched back to the chow diet, resulting in body weights similar to those of CTRL mice at the beginning of each new WD cycle (Figure 1B). Consequently, although mice continuously maintained on WD gained more weight than CYCL mice between the different WD cycles, by the end of cycles 2 and 3, CYCL mice had caught up with the WD mice in terms of weight gain (Figure 1B).

In response to each WD introduction, mice showed a 24‐hours hyperphagic phase, which was followed by normalization of their energy intake within the five following days (Figure 1C). This normalization was sustained throughout the experiment in mice continuously fed WD (Figure S2, Supporting Information). On the contrary, CYCL mice exhibited significant decrease in daily energy intake when they were switched back to the chow diet, compared to the intake on the last day of WD (Figure 1C). The energy intake during the 24‐hours hyperphagic phase upon WD introduction did not differ among WD cycles. However, when we evaluated the WD consumption pattern during the first hours following WD introduction, we observed a major increased intake during the first 8 h following WD reintroduction (Figure 1D, **left panel**). Noteworthy, WD was introduced in home cages 2hours after the onset of the light period, when mice are usually at rest, suggesting that CYCL mice displayed a period of heightened hedonic appetite during the light phase. Moreover, WD consumption during these first 8 h increased with the number of cycles, with a significant 32% increase between cycles 1 and 3 (Figure 1D). This incremental increase in WD consumption was specific to the CYCL mice, as this pattern completely disappeared after the first exposure to the WD diet in mice maintained continuously on WD and was not observed in the CTRL mice (Figure 1D, **left panel**). By day 3, no differences in diurnal or nocturnal caloric intake were observed between groups, suggesting that the CYCL‐enhanced body weight gain did not result from a disruption of the circadian cycle of food intake (Figure S3, Supporting Information).

### 2.2 Weight Cycling Induces Remodeling of Central Structures Involved in Food Intake Control

**Figure 2:**
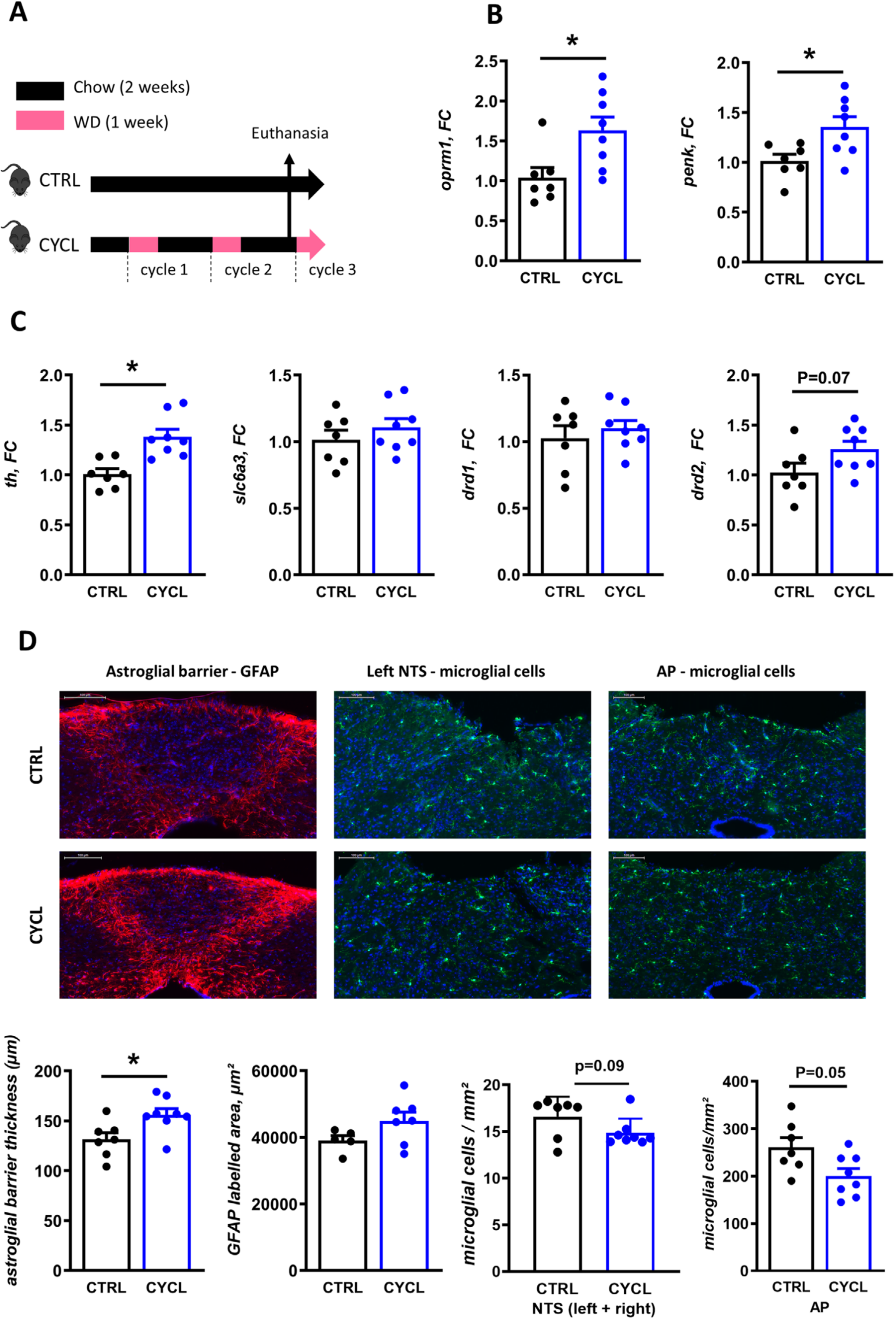
CYCL mice under chow diet display heightened reward phenotype in the striatum and altered barrier properties in the dorso‐vagal complex. A) Experimental design: Mice were submitted to cycles of one week of western diet (WD) followed by 2 weeks of chow diet (CYCL mice) or were left under chow diet (CTRL). They were euthanized while fed chow diet one day before the beginning of the third WD cycle. Age‐matched CTRL mice fed only chow diet were euthanized at the same time. B) Relative expression of genes involved in the opioïdergic system (*oprm1*: Opioid Receptor Mu 1 and *penk*: proenkephalin) in the striatum of CTRL and CYCL mice under chow diet. C) Relative expression of genes involved in the dopaminergic system (*th*: tyrosine hydroxylase, *slc6a3*: dopamine transporter 1, *drd1*: dopamine receptor 1 and *drd2*: dopamine receptor 2) in the striatum of CTRL and CYCL mice under chow diet. D) Astroglial barrier thickness between the area postrema (AP) and the nucleus tractus solitarius (NTS), astroglial cell (glial fibrillary acidic protein (GFAP)–labelled) spreading between AP and NTS and microglial cell density in the AP and in the NTS (left and right) of CTRL and CYCL mice under chow diet. Representative images are presented above graphs. *n* = 7‐8 mice/group, *p < 0.05 (Mann‐Whitney test). Each dot represents one mouse. Bars are means ± SEM.

Eating behavior is regulated by the integration of signals originating from the gut (nutrients, bacterial metabolites) at different levels of the central nervous system, in particular in the dorso vagal complex (DVC), the hypothalamus, and the striatum. Therefore, we evaluated the impact of recurrent exposure to WD on these different structures, by collecting samples from CYCL‐chow mice the day before WD was reintroduced for the third time and comparing them to age‐matched CTRL mice (**Figure** **2**A).

In the striatum, dietary alternation induced alterations in both the opioidergic and dopaminergic systems in CYCL mice compared to CTRL mice, both under the chow diet. These changes were characterized by a significant increase in gene expression of the opioid receptor mu 1 (*oprm1*) and proenkephalin (*penk*) (Figure 2B) as well as tyrosine hydroxylase (*th*), the rate‐limiting enzyme in the biosynthesis of catecholamines, including dopamine. Additionally, there was a trend toward increased expression of the dopamine receptor 2 (*drd2*) (Figure 2C) in CYCL mice. Expression assay in the whole hypothalamus did not reveal any change in the relative expression of genes involved in regulation of food intake, plasticity, blood‐brain barrier, astrocyte and microglia markers or inflammatory markers following dietary alternation (**Table** S1, Supporting Information).

In the DVC, the area postrema (AP) hosts a rich population of microglia and is bordered by a thick barrier of astroglial cells that may control the transfer of circulating molecules from the AP to the blood‐brain barrier‐protected nucleus of the DVC. These glial populations have been shown to react to WD^[^ ^21^, ^22^ ^]^ In our study, the deployment of the astroglial barrier between the AP and the nucleus tractus solitarius (NTS) was increased, showing a larger GFAP‐labelled surface and thickness, in CYCL than in CTRL mice (Figure 2D). The density of microglial cells within the AP and NTS tended to be lower (P = 0.054) in CYCL compared to CTRL mice (Figure 2D). Despite these cellular changes observed in histological DVC slices, we did not observe any difference in the expression of genes related to regulation of food intake, plasticity, blood‐brain barrier or microglia, astrocytes, and inflammation between CTRL and CYCL mice in AP samples isolated by dissection from the brainstem (**Table** S1, Supporting Information).

### 2.3 Weight Cycling Modifies Caecal Microbiota under Chow Diet

**Figure 3:**
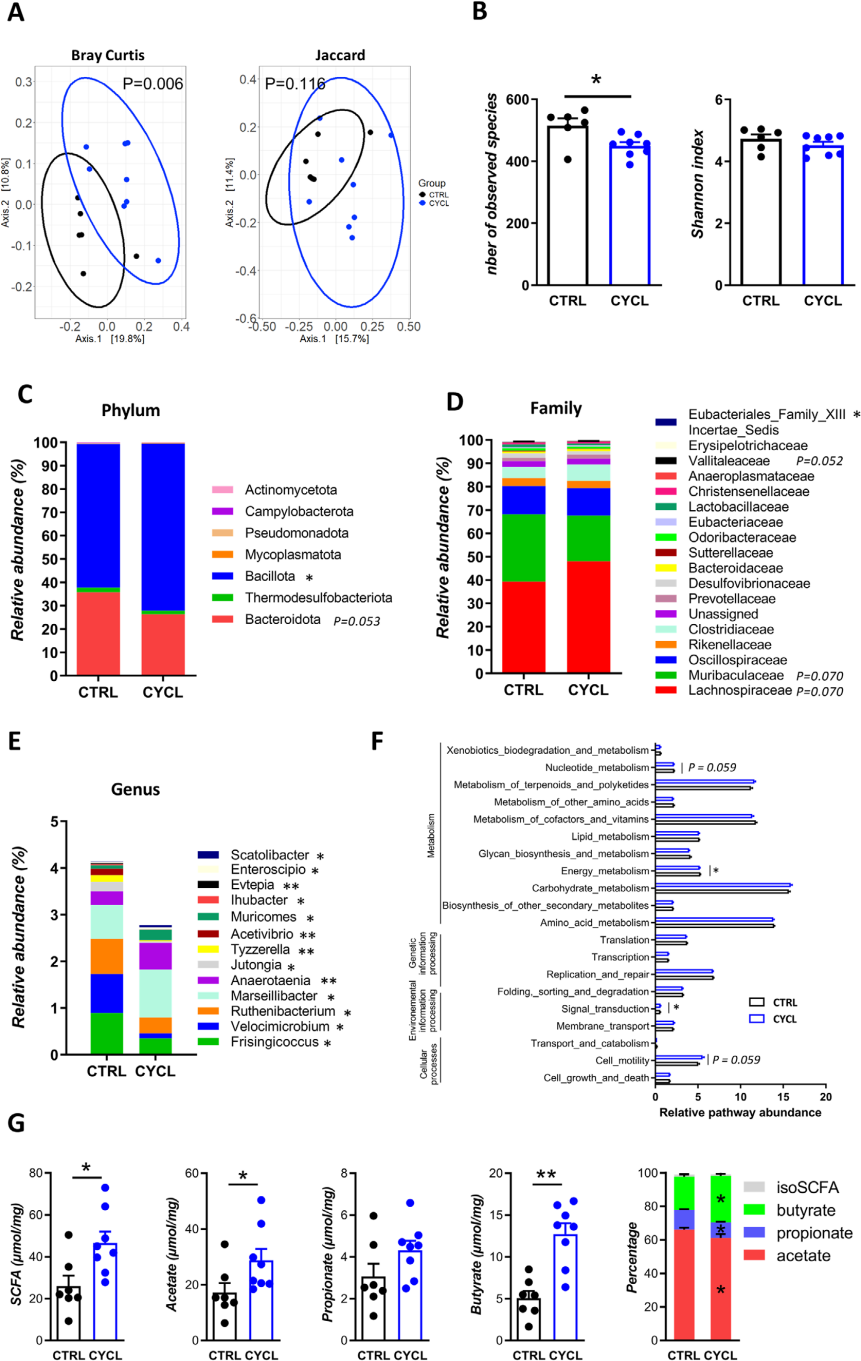
CYCL mice display caecal dysbiosis under chow diet. A) Principal Coordinates Analysis using Jaccard and Bray‐Curtis distances of the caecal microbiota of CTRL and CYCL mice under chow diet. B) Number of observed species and Shannon index of the caecal microbiota of CTRL and CYCL mice under chow diet. C) Relative abundances of the different phyla in the caecum of CTRL and CYCL mice under chow diet. D) Relative abundances of the 19 most abundant families in the caecum of CTRL and CYCL mice under chow diet. E) Relative abundances of the 13 genera with significantly abundant in the caecum of CTRL and CYCL mice under chow diet. F) Relative abundance of the predicted Level 2 KEGG metabolic pathways in the caecum of CTRL and CYCL mice under chow diet. G) Caecal total and main short‐chain fatty acids (SCFA) concentration and relative proportions of the different SCFA in the caecum of CTRL and CYCL mice. *n* = 7‐8 mice/group, *p < 0.05 (Mann‐Whitney test). Each dot represents one mouse. Bars are means ± SEM.

Yoyo dieting has already been shown to induce intestinal dysbiosis, even after mice have returned to chow diet.^[^ ^12^ ^]^ Therefore, we evaluated if the microbiota of CYCL mice was altered by the alternation of WD and chow diets by analyzing the caecal composition of CTRL and CYCL mice under chow diet. Caecal dysbiosis was manifest in CYCL‐chow mice when compared to CTRL‐chow ones as demonstrated by the principal coordinate analysis using Jaccard distance (**Figure** **3**A). The number of observed species was lower in CYCL than CTRL mice but evenness, showed by the Shannon index, was similar between groups (Figure 3B). Caecal dysbiosis in CYCL‐chow mice extended to taxonomic differences at multiple levels compared to CTRL‐chow mice (Figure 3C–E). At the phylum level, Bacillota was significantly enriched in CYCL mice, whereas Bacteroidota showed a trend toward lower abundance (p = 0.053) (Figure 3A). At the family level, Eubacteriales_Family_XIII_Incertae_Sedis was significantly reduced in CYCL compared to CTRL mice, and Vallibacteraceae as well as more abundant families such as Muribaculaceae, and Lachnospiraceae also exhibited a tendency to differ between groups (0.05 ≤ P ≤0.07) (Figure 3D). At the genus level, a subset of 13 low‐abundance genera (<1%) was significantly affected by weight cycling (Figure 3E; **Table** S2, Supporting Information). The analysis of the predicted functional composition of the metagenome at a general level (level 2 of (Kyoto Encyclopedia of Genes and Genomes) KEGG pathways, Figure 3F) highlighted the importance of carbohydrate metabolism in the gut microbiota of both CYCL and CTRL mice. Among the metabolic functions, while carbohydrate, lipid, and amino acid metabolism were not significantly affected by weight cycling, PICRUSt2 analysis revealed a significant decrease in energy metabolism and a trend toward decreased nucleotide metabolism pathways in CYCL mice compared to CTRL mice. Additionally, there was a significant increase in signal transduction and a trend toward higher cell motility pathway relative abundance in CYCL mice. At a more precise level (level 3 of KEGG pathways, Suppl. **Table** S3), several metabolic pathways were reduced in the microbiota of CYCL mice, including the tricaboxylic acid (TCA) cycle, ubiquinone and other terpenoid‐quinone biosynthesis pathways, carbon fixation, inositol phosphate metabolism, consistent with the reduced energy metabolism pathway observed at a higher pathway level. Other pathways, such as flavonoid biosynthesis, vitamin B6 metabolism, and sulfur metabolism were reduced in the microbiota of CYCL mice (**Table** S3, Supporting Information).

Given that carbohydrate metabolism was the most abundantly represented and contributes, among other things, to the production of SCFA, we evaluated the SCFA concentration in the caecum of both groups of mice. The total amount of SCFA was significantly greater in CYCL mice compared to CTRL ones, despite the fact that both groups were fed the same chow diet (Figure 3G). This difference might be attributed to a significant increase of acetate and butyrate levels, but not propionate. Consequently, the relative proportions of the major SCFAs was altered with significant lower proportions of acetate and propionate but a greater proportion of butyrate in CYCL compared to CTRL mice (Figure 3G). These results confirm that cycling between WD and chow diet induce metabolic alterations of the bacterial community.

### 2.4 Transferring the Microbiota of Mice that Underwent Weight Cycling Transferred their Transient Heightened Hedonic Appetite

**Figure 4:**
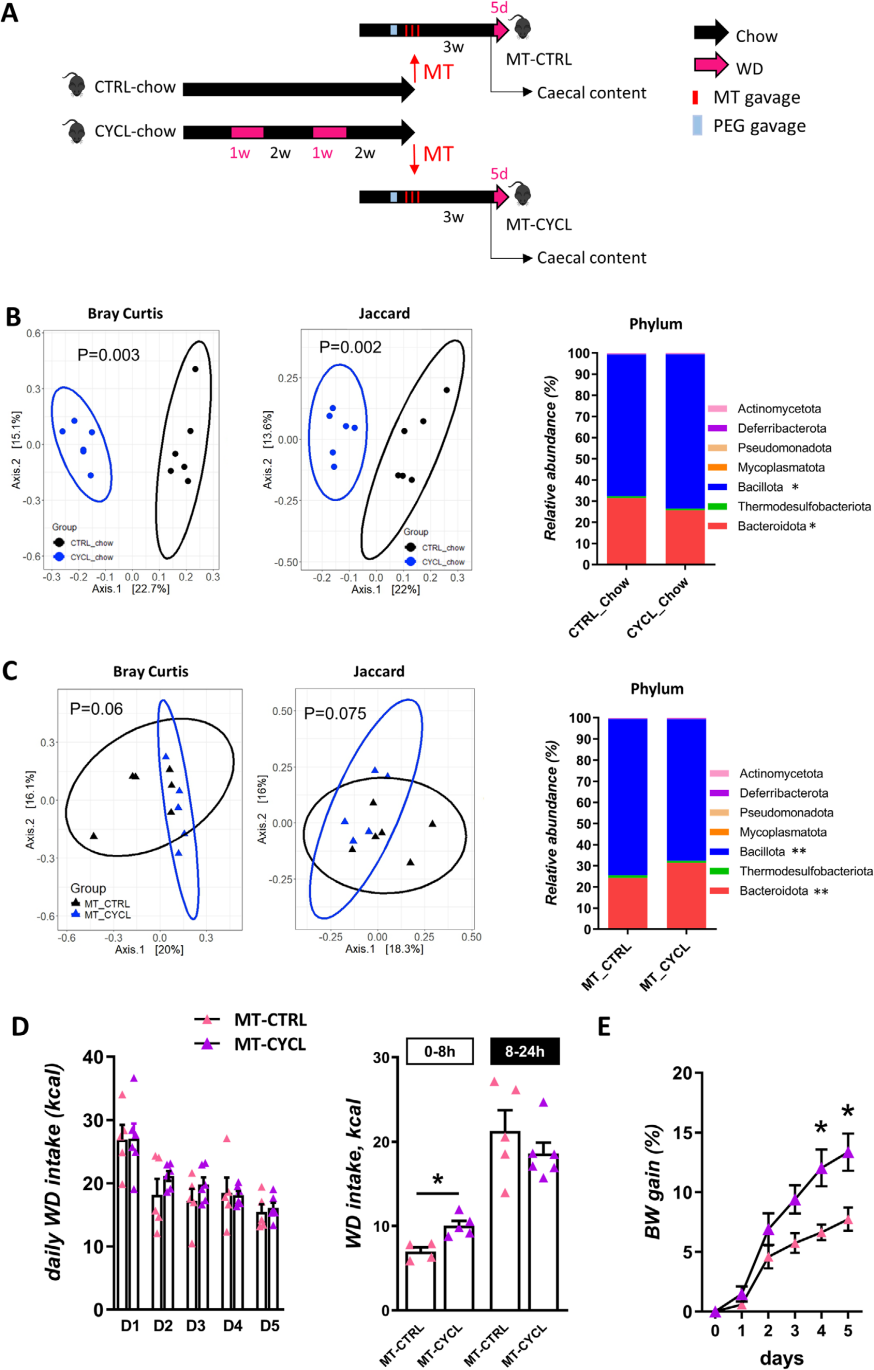
Transferring CYCL‐chow microbiota to naïve mice transfers behavioral phenotype. A) Experimental design: Donor mice were submitted to cycles of one week of western diet (WD) followed by 2 weeks of chow diet (CYCL) or left under chow diet (CTRL). They were euthanized under chow and their caecal microbiota transferred on 3 consecutive days (MT transfer) to recipient mice (MT‐CYCL and MT‐CTRL, respectively) that had been previously prepared by polytethylene glycol (PEG) gavage. Recipient mice were left undisturbed under chow diet for 3 weeks before being either euthanized or switched to WD for 5 days. B) Principal coordinate analysis based on Jaccard and Bray‐Curtis distance and relative abundance of phyla of CTRL and CYCL donor mice. C) Principal coordinate analysis based on Jaccard and Bray Curtis distances and relative abundance of phyla in the caecal microbiota of recipient mice (MT‐CTRL and MT‐CYCL) 3 weeks after microbiota transfer. D) Daily energy intake after WD introduction and energy intake the first 8 and following 16 h after the switch to WD. E) Body weight gain after WD introduction in recipient mice. *n* = 5‐6 mice/group *p < 0.05 and **p < 0.01 (Mann‐Whitney test). Each dot represents one mouse. Bars are means ± SEM.

The role of the microbiota in regulation of palatable food intake has been recently described^[^ ^23^, ^24^, ^25^ ^]^ We thus sought to evaluate if the dysbiosis induced by weight cycling could be at play in the transient heightened hedonic appetite we observed in CYCL mice. Thus, we transferred the caecal microbiota of CYCL mice that had underwent 2 cycles of diet alternation (MT‐CYCL) or of age‐matched CTRL mice to naïve recipient mice (MT‐CTRL) (**Figure** **4**A). We confirmed that the caecal microbiota of donor mice differed, with a significant increase in Bacillota and a significant decrease in Bacteroidota relative abundances in donor CYCL mice compared to CTRL ones (Figure 4B). Three weeks after transfer, the microbiota of MT‐CTRL and MT‐CYCL mice tended to differ and the similar differences in phylum relative abundance (a significant increase in Bacillota and a significant decrease in Bacteroidota) observed in the donor mice were observed in the recipient ones (Figure 4C).

We evaluated the eating behavior response to WD introduction of transferred mice by exposing them to WD for 5 days. As expected, all mice exhibited a first 24–hours‐hyperphagia period upon WD introduction, that normalized after 1 day, irrespective of their group (Figure 4D). When we examined more precisely the WD intake during the first 8 h following WD introduction, MT‐CYCL mice ate significantly more WD than MT‐CTRL ones (Figure 4D), suggesting a heightened hedonic appetite, as observed in donor mice. Moreover, as observed in donor mice, body weight gain during the 5 days under WD was greater in MT‐CYCL mice than MT‐CTRL ones (Figure 4D). Noteworthy, eating behavior and body weight gain of transferred mice did not differ under chow diet before WD introduction (Figure S4, Supporting Information).

## 3 Discussion

This study demonstrates that alternating between high‐energy/palatable and standard diets, a model of yo‐yo effect of recurrent restrictive dieting, remodels the gut microbiota‐brain axis toward a profile that is associated with heightened hedonic appetite and risk of weight gain. Moreover, transferring the microbiota of weight cycling mice to naïve ones never exposed to WD recapitulates this heightened hedonic appetite toward WD, indicating the involvement of this dysbiotic microbiota in the altered eating behavior observed upon weight cycling.

To detect potential modifications in eating behavior, we chose to study weight cycling in a mouse model with ad libitum access to food. In this model, we show that mice gain and lose weight during WD and chow periods, respectively. In addition, each dietary switch was accompanied by opposite food intake adaptations. A period of transitory hyperphagia was observed during the transition from the chow diet to WD, while a transient hypophagia was measured when switching from WD back to the chow diet. The WD, which is high in sugar and fat, is a highly palatable diet able to stimulate food intake even in the absence of hunger, leading to food overconsumption^[^ ^26^, ^27^ ^]^ Mice, like other rodents, display neophobia and will normally avoid novel foods. However, enhanced caloric intake upon first time WD exposure has been observed in several studies, by our group^[^ ^15^, ^19^, ^28^ ^]^ The palatability of WD and activation of the mesolimbic system^[^ ^29^ ^]^ classically explain the increase in food consumption when transitioning to WD. Importantly, Benani et al. tested the role of novelty in this hyperphagic behavior and observed that mice ate the same amount of food in 24hrs whether they were submitted once or twice to a 1‐day WD period, with a one‐week period of chow diet in between.^[^ ^28^ ^]^ This suggests that there is no effect of novelty in this behavior. After a few days on WD, food intake normalizes likely due in part to hypothalamic rewiring of POMC neurons.^[^ ^28^ ^]^ Finally, when switching from WD to chow diet, transient hypophagia has been previously observed and was associated with changes in hypothalamic and mesolimbic activity^[^ ^30^, ^31^ ^]^ leading to the devaluation of a less palatable diet, reducing the consumption of chow diet. Determining whether these changes in food intake patterns when switching diets are dependent upon specific diet durations is difficult to establish. Devaluation of chow diet after WD withdrawal has been constantly observed, irrespective of the duration of the WD period,^[^ ^31^ ^]^ suggesting that the same reduction in chow diet consumption would have been observed even with a different WD duration in our study. On the other hand, in studies that used paradigm of high‐energy/palatable and standard diet alternation to mimic weight cycling in rodents, some authors reported an increase in WD consumption,^[^ ^13^ ^]^ while other did not find this effect^[^ ^12^, ^32^ ^]^ In these studies, the duration of the specific diets was highly variable (2‐weeks / 2‐weeks (13), 8‐weeks/8‐weeks^[^ ^32^ ^]^ or variable durations of WD and chow diet exposures to ensure CYCL mice return to the control group average body weight during weight loss or to the obese mice group average body weight during weight regain.^[^ ^12^ ^]^ In these different studies, WD intake was reported on a daily or weekly basis but the authors did not finely evaluate caloric intake in the first hours following WD introduction, preventing any firm conclusion on whether diet duration could influence our results.

Interestingly, in our study, WD‐induced hyperphagia progressively intensifies during the first few hours of each cycle of WD introduction. This pattern suggests that components of food reward (i.e. liking, wanting, and learning) may be affected in our model. Although this hyperphagic behavior could be attributed to a learned anticipation of WD, as seen in models of intermittent access to palatable food^[^ ^33^, ^34^ ^]^ the second introduction of WD occurred at a time that could not be predicted by the animals. It is therefore unlikely that this behavior reflects an anticipation of WD learning The progressive increase in behavioral response to the palatable diet upon re‐exposure is reminiscent of behavioral sensitization observed with repeated drug use^[^ ^35^, ^36^ ^]^ and may indicate an increased reinforcing value of the WD reward. Interestingly, the drug incentive‐sensitization theory is proposed to explain binge‐eating disorders, where excessive eating results from excessive food wanting without necessarily increased liking^[^ ^35^, ^37^, ^38^ ^]^ Furthermore, withdrawal of WD has been shown to elevate stress,^[^ ^39^ ^]^ a state known to exacerbate food wanting and mesolimbic reactivity.^[^ ^40^, ^41^ ^]^ Therefore, repeated WD withdrawal in our model might progressively enhance WD wanting. Finally, despite the escalation in energy intake during the first few hours of renewed WD access, the total energy consumption of the CYCL mice over the first 24–hours remained stable over the successive cycles. These data suggest that although food reward valuation and wanting processes might be altered in this model, the homeostatic regulation of food intake is still functional. Therefore, weight maintenance during restrictive yoyo dieting might be impeded not only by metabolic adaptations but also by modified food reward‐related processes. This is further emphasized by the increased expression of genes involved in the opioidergic and dopaminergic system in the striatum of CYCL mice; a cerebral component of the reward system, where these neuromodulatory systems play a crucial role in liking and wanting of food reward.^[^ ^42^, ^43^, ^44^, ^45^ ^]^

In the current study, we found that not only the striatum but also the astroglial barrier between the AP and NTS was affected by alternation between WD and chow diets. The AP and NTS, located in the DVC of the brainstem, integrate signals from the gut and facilitate communication with brain structures involved in eating behavior.^[^ ^46^ ^]^ In this study, we evidenced for the first time a long‐lasting effect of WD on astrocyte deployment in the brainstem,^[^ ^47^ ^]^ despite the mice being on a chow diet for the last two weeks. The thicker astroglial barrier in CYCL mice likely serves as a protective mechanism against circulating peripheral signals entering the AP, where the blood brain barrier is leaky.^[^ ^48^ ^]^ Interestingly, we noted a decrease in microglial cell density in both the AP and the NTS of CYCL mice fed the chow diet. Generally, microglial density increases in response to WD exposure, indicating a pro‐inflammatory situation.^[^ ^49^ ^]^ The reduced number of microglial cells may be related to the marked decreased food intake observed in CYCL mice during the second cycle of chow diet. This lower intake likely led to a reduced influx of circulating metabolites, such as fatty acids, which may have tempered the microglial presence and activation within the AP.

The transfer of gut microbiota from CYCL mice to naive recipient mice (naive for WD exposure) reproduced the altered eating behavior upon WD exposure, demonstrating that microbiota changes induced by recurrent restrictive dieting play a causal role in hedonic feeding regulation. Because of this important demonstration of the role of the microbiota, we further explored the potential bacteria, bacterial metabolites, or metabolic pathways involved in this process. In our model, gut microbiota of weight cycling mice was impoverished, a consistent characteristic of WD‐chronically fed individuals. Since MT‐CYCL mice show heightened hedonic appetite, this could suggest that an impoverished gut microbiota is associated with altered food reward processes. Indeed, several preclinical studies using microbiota transfer from WD‐chronically fed rodents established that these impoverished gut microbiotas alter reward signaling, motivational drive, and food intake^[^ ^50^, ^51^, ^52^ ^]^ highlighting the regulating role of gut microbiota on hedonic feeding.^[^ ^24^ ^]^ At the phylum level, our data indicate that weight cycling leads to an increased abundance of Bacillota (ex‐Firmicutes) at the expense of Bacteroidota. This increase in Bacillota relative abundance was measured in CYCL mice under chow before switching to WD as well as in MT‐CYCL. Such change in Bacillota / Bacteroidota ratio is commonly observed in human and mouse models of obesity^[^ ^53^ ^]^ and increased abundance of Bacillota is associated with increased energy harvesting and fat storage in the host.^[^ ^54^, ^55^ ^]^ The predicted functional composition of the metagenome indicated reduced bioenergetic metabolism in the microbiota of CYCL mice, with among the different predicted pathways, a reduction in TCA cycle and ubiquinone biosynthesis pathways. This suggests reduced energy utilization by the gut microbiota, likely to the benefit of the host. The Bacillota phylum contains many bacteria recognized as SCFA producers^[^ ^56^, ^57^ ^]^ consistent with our data where total SCFA was increased in the caecum of CYCL mice compared to CTRL ones. Besides being energy source for the host, SCFAs are also bacterial‐derived metabolites known to regulate appetite and reward‐related behaviors.^[^ ^23^, ^58^, ^59^, ^60^ ^]^ Hence, the overall increase and/or change in proportions of the main SCFAs may contribute to the heightened hedonic appetite observed in CYCL mice. The relative proportions of the major SCFAs were altered, with lower proportions of acetate and propionate but a greater proportion of butyrate in CYCL compared to CTRL mice. Bacteria belonging to the Bacteroidota phylum produce mostly acetate and propionate, and those of the Bacillota phylum have butyrate as their main fermentation end product.^[^ ^61^ ^]^ Hence these results are consistent with the increased Bacillota/Bacteroidota ratio we observed. Acetate can directly suppress appetite via its action on the brain^[^ ^62^ ^]^ and propionate has been linked to appetite reduction and improved weight management in overweight adults through stimulation of GLP‐1 release.^[^ ^63^ ^]^ In CYCL mice, while relative levels of propionate and acetate were decreased, absolute level of propionate was unchanged and that of acetate was increased in comparison with control mice. Butyrate, which improves insulin sensitivity, exhibits anti‐inflammatory effects, and is generally associated to health benefits,^[^ ^56^ ^]^ showed both relative and absolute increases after weight cycling in our model. Given these findings, it remains challenging to determine whether the food intake dysregulation in CYCL mice is driven by the overall increase in total SCFA, changes in acetate/propionate/butyrate ratio or specific alterations in one of these SCFAs.

Besides changes in energy metabolism and SCFA concentrations, other predicted metabolic pathways were modified in CYCL mice compared to CTRL ones. Flavonoid biosynthesis pathway was reduced in mice that underwent weight cycling, consistent with the reduced flavonoids levels observed in another weight cycling study.^[^ ^12^ ^]^ The role of flavonoids, on brain health has been uncovered in the last decade,^[^ ^64^ ^]^ including in behaviors involving reward such as alcohol use disorders^[^ ^65^ ^]^ or nicotine‐induced reward.^[^ ^66^ ^]^ Moreover, the sulfur metabolism pathway was reduced in CYCL compared to CTRL mice. The end‐product of this pathway is H2S that can exert beneficial or detrimental effects of the host epithelium, depending on its intraluminal concentration.^[^ ^67^ ^]^ A dual role of H2S on the gut‐brain axis is suggested by several studies,^[^ ^68^, ^69^, ^70^ ^]^ through its anti‐inflammatory effects or by stimulating the vagus nerve. Whether changes in these two metabolic pathways, in addition to the increased SCFA levels participate to the heightened hedonic appetite would need further research.

Finally, at the genus level, in mice with a history of weight cycling the abundance of several bacteria was significantly decreased (*Acetivibrio, Evtepia, Frisingicoccus, Ihubacter, Jutongia, Ruthenibacterium, Tyzzerella, Velocimicrobium*) and of others increased (*Anaerotaenia, Enteroscipio, Marseillibacter, Muricomes*) compared to CTRL mice. While some of these bacteria have been associated in the literature with brain disorders, including depression and addiction^[^ ^71^, ^72^, ^73^, ^74^, ^75^, ^76^, ^77^, ^78^ ^]^ no data are currently available on the link of these genera with eating behavior. Interstingly, among these genera, *Ruthenibacterium* was highly increased in the feces of healthy volunteers under a complete fasting 10‐days experimental program and was shown to mitigate high fat diet induced obesity in mice,^[^ ^79^ ^]^ suggesting a role in lipid metabolism. The reason why we observed a decreased relative abundance of *Ruthenibacterium* in the caecum of CYCL mice at the end of the weight loss period is currently unclear but we speculate dynamic changes of this genus abundance depending upon the intensity of the weight loss, which was more intense during the first week compared to the second week under chow diet. Hence, at this stage, the role played in the behavioral phenotype of CYCL mice by this genus and the other ones, for whom almost no data are available, would be highly speculative. In addition, the relatively limited overlap in microbial profiles at the genus level between mice and humans^[^ ^80^ ^]^ is important to acknowledge in the context of translating these results to human health. Hence, further work is thus warranted to understand whether this remodeling at the genus level modifies significantly host physiology and behavior and its relevance to human health.

To conclude, in this study we showed that alternation between high‐energy and standard diet durably remodels the gut microbiota toward a profile that is associated with an increase in hedonic appetite and weight gain. Using gut microbiota transfer, we established that this yoyo microbiota signature affects hedonic appetite. More work is definitely needed to fully understand the mechanisms at play in this model, especially regarding the gut microbiota to brain transduction pathways involved in this weight cycling‐induced altered eating behavior. The gut microbiota can influence brain and reward processes through multiple pathways, including neural (via the vagus nerve and spinal afferents), humoral (involving, intestinal hormones, gut‐derived endocannabinoids, neurotransmitters produced by bacteria from key amino acid precursors or other microbial metabolites), and immune pathways (modulating neuroinflammation and gut permeability).^[^ ^23^, ^81^ ^]^ Notably, recent data have highlighted the role of microbiota‐induced neuroinflammation in the dysregulation of the reward system following chronic exposure to WD.^[^ ^82^ ^]^ The activation of the vagus nerve and its influence on the reward system, including striatal neuron activation, has also been documented.^[^ ^51^ ^]^ Brain plasticity, including vagal rewiring potentially associated with microglial changes in the NTS, is also likely at play, although no difference in the expression of different genes involved in plasticity was noticed in the AP and the hypothalamus between CYCL and CTRL mice.

There is ongoing concern that dietary interventions used for weight management may contribute to the development of eating disorders, which motivated research initiatives, such as the Eating Disorders In weight‐related Therapy (EDIT) Collaboration, that investigates this issue through different approaches in Humans.^[^ ^83^ ^]^ Our data, highlighting the possible role of the gut microbiota in this phenomenon, suggest new directions that could be evaluated in Humans and are therefore highly complementary to these initiatives. However, we believe that this work adds to the comprehension of the interplay between gut microbiota, eating behavior and weight management as collectively, these data highlight a putative microbiota signature in individuals prone to weight gain and at risk for hyperphagia of palatable food.

This study has several limitations that should be considered when interpreting the results. First, we did not implement operant tests to measure reward‐related motivational and learning aspects, nor did we assess the hedonic impact of food through taste reactivity tests. Given that changes in the reinforcing value of food are known to predict weight changes in humans^[^ ^84^ ^]^, future research should focus on different components of reward, particularly wanting, mesolimbic reactivity, and anxiety within this model to provide a more comprehensive understanding. Our study underscores the major influence of gut microbiota on behavior, thus, these additional tests should be carefully designed to avoid confounding effect of using food reward in behavioral tests or any procedure that might impact gut microbiota. Additionally, the study was conducted exclusively on male mice, which limits the generalizability of the findings to females. Recent studies have shown that male and female mice respond differently to WD exposure, with females exhibiting more pronounced hypothalamic glial cell activation and a greater increase in Pomc gene expression compared to males.^[^ ^85^, ^86^ ^]^ Other research has also highlighted sex‐dependent effects of WD on intestinal gut microbiota composition. Future studies should explore these sex‐specific differences to better understand the impact of weight cycling in both sexes. Lastly, while this study examined the behavioral effects of a ‘yo‐yo’ microbiota, further research is needed to investigate the cerebral substrates underlying this phenotype after microbiota transfer.

## 4 Experimental Section

### Animals

The present protocols received written agreement from the local ethic committees: Comité Rennais d'Ethique en matière d'Expérimentation Animale (CREEA) and Comité d'éthique local pour l'expérimentation animale du centre de recherches de *Jouy en Josas* (*COMETHEA*) (file no. APAFIS#40563‐2023013010327051v2 for experiments 1 and 2, and APAFIS#2020051821292405 for experiment 3). The experiments comply with the ARRIVE guidelines and have been carried out in accordance with the EU Directive 2010/63/EU for animal experiments. For all experiments, 7‐week old mice were housed individually at arrival and fed a regular chow diet (chow, ref V1124‐000, SSniff, Germany) ad libitum under a 12:12 hours light/dark cycle (22 ± 2°C). They were acclimatized to the animal facility for 1 week. Western Diet (WD, ref RD12451, Research diet, USA) was introduced at the beginning of the light cycle (resting period) for each cycle. Mice were euthanized during the first half of the light cycle by cervical dislocation.

Experiment 1. C57Bl6/N male mice (Janvier labs, France) were pseudo‐randomized based on their body weight into three homogenous groups: CYCL mice underwent 3 cycles of 1 week of WD feeding each followed by 2 weeks of chow diet feeding (*n* = 6), except on cycle 3 were mice were left on WD to evaluate the kinetic of weight rebound. All diets were provided ad libitum. CTRL mice received chow diet during the whole experiment (*n* = 6) while WD mice received WD diet during the whole experiment (*n* = 6). Food intake was measured for the 3 groups during the first 8 h following WD introduction (9AM to 5PM) and the subsequent 16–hours (5PM to 9AM the next day) over three days for each of the 3 WD cycles in order to evaluate hedonic and homeostatic appetite based on a previous study that demonstrates that the overconsumption of palatable food is limited to the first half of the light phase.^[^ ^20^ ^]^ Food intake and body weight were also measured daily during all the experiment. Fecal samples were collected at different time points along the experiment. Experimental protocol is summarized in Figure 1A. Food consumption was expressed as caloric intake normalized to body weight or basal food intake.

Experiment 2 was conducted in two sets. In the first set, C57Bl6/N male mice (Janvier labs, France) were pseudo‐randomized based on their body weight into two homogenous groups: CTRL mice were fed the chow diet ad libitum. CYCL mice underwent 2 cycles of 1 week of WD feeding each followed by 2 weeks of chow diet and were euthanized at the end of the 2nd chow period (*n* = 7–8 for each group). Experimental protocol is summarized in Figure 2A. Food intake was measured daily during the whole experiment. Caecal content and striatum were stored at −80°C after sampling for later 16S RNA and gene expression analysis respectively. Brainstems were dissected, fixed in 10% neutral buffered formalin (formaldehyde 4%, Diapath) for 24–hours at 4°C, cryoprotected with sucrose (30% in PBS), frozen in isopentane (−40°C), then coronal sections were performed on a cryostat (Leica CM3050S) and collected on slides for immunohistochemistry. The second set followed the same design with *n* = 7 for each group. The hypothalamus and area postrema (AP) of the brainstem were dissected and stored at −80°C after sampling for later gene expression analysis.

Experiment 3. *Donor mice (n = 6 per group)*: C57Bl6/N male mice (Envigo, France) were pseudo‐randomized based on their body weight into two homogenous groups: CTRL mice were fed a chow diet during all the experiment and CYCL mice underwent 2 cycles of 1 week of WD consumption each followed by 2 weeks of chow diet consumption. Mice were euthanized at the end of the 2nd chow period. *Preparation of the inoculum*: Caecal contents were sampled from donor mice at the time of euthanasia, diluted at a 1:50 (mass:volume) ratio in maltodextrin‐trehalose diluent,^[^ ^87^ ^]^ aliquoted and transferred to recipient mice or stored at −80°C. *Recipient mice (n = 12 per group)*: C57Bl6/N male mice (Envigo, France) were gavaged 5 times within a 2‐h period with 200µL of a polyethylene glycol (PEG) solution (Macrogol, 4000, 425g L^−1^ in sterile water) to deplete their gut microbiota. They were then divided into 2 groups of weight‐matched microbiota‐transferred (MT) mice (MT‐CTRL and MT‐CYCL). Four hours after the last PEG gavage, they were colonized by intra‐gastric gavage of 200 µL of inoculum prepared from CTRL and CYCL mice, respectively. One donor mouse colonized 2 recipient mice. The next two days after transfer, recipient mice were gavaged with 200µL of frozen/thawed of the same inoculum. Body weight and food intake of recipient mice was monitored every week. Three weeks after inoculation, half of the recipient mice were euthanized to collect caecal contents. The daily food intake of the second half of the mice was monitored during the last 24–hours under chow diet and during 5 days after a switch to WD. Every day food intake was measured between 9am and 5pm (0–8h) and during the following 16 h (8–24h). Experimental protocol is summarized in Figure 4A.

### Determination of Caecal Microbiota Composition

Total DNA was extracted from caecal content samples using the ZR fecal DNA Miniprep (Ozyme). The V3‐V4 region of DNA coding for 16SrRNA was amplified using the following primer: CTTTCCCTACACGACGCTCTTCCGATCTACTCCTACGGGAGGCAGCAG (V3F) and GGAGTTCAGACGTGTGCTCTTCCGATCTTACCAGGGTATCTAATCC (V4R), Taq Phusion (New England Biolabs) and dNTP (New England Biolabs) during 25 cycles (10 s at 98°C, 30 s at 45°C, 45s at 72°C). Purity of amplicons was checked on agarose gels before sequencing using Illumina Miseq technology, performed at the Genotoul Get‐Plage facility (Toulouse, France). Sequences are available under https://doi.org/10.57745/OK3ZHQ. Raw sequences were analyzed using the bioinformatic pipeline FROGS.^[^ ^88^ ^]^ Amplicon Operational Taxonomic Unit (OTU) were clustered using Swarm with parameter d = 1 and chimeras were filtered following FROGS version 4.1 guidelines. Assignation was performed using 16S_REFseq_Bacteria_20230726 database. OTU with abundances lower than 0.0005% of the total read set were removed prior to analysis. Subsequent analysis were done using the phyloseq R packages.^[^ ^41^, ^89^, ^90^ ^]^ Samples were rarefied to even sampling depths before computing within‐samples compositional diversities (observed richness and Shannon index) and between‐samples compositional diversity to performed Multidimensional Scaling (MDS) analysis using Bray‐Curtis or Jaccard dissimilarity. Raw unrarefied OTU counts were used to produce relative abundance graphs. The predictive functional profiling of bacteria communities was analyzed using the Phylogenetic Investigation of Communities by Reconstruction of Unobserved States 2 (PICRUSt v2.5.0)^[^ ^89^ ^]^ against KEGG database.^[^ ^91^ ^]^

### Determination of Caecal SCFA Levels

SCFA caecal levels were determined as previously described.^[^ ^92^ ^]^ Briefly, SCFA were water‐extracted and proteins were precipitated with phosphotungstic acid. Supernatant (containing SCFA) was analyzed in duplicate using gas‐liquid chromatography (Auto‐system XL; Perkin Elmer, Saint‐Quentin‐en‐Yvelines, France). Peaks were integrated using the Turbochrom v6 software (Perkin Elmer, Courtaboeuf, France).

### Brainstem Glial Immunohistochemistry

Immunohistochemistry (IHC) was performed on 20‐µm thick serial horizontal brainstem sections containing the AP and tractus solitarius nucleus (NTS) to evaluate microglial and astroglial morphology. Non‐specific sites were blocked by incubation with bovine serum albumin (BSA) (2% in PBS 0.1M; Triton X‐100 0.3%) for 2 hours at room temperature. Astrocytes were labeled for GFAP by incubating the slices for 1 h with a monoclonal Cy3‐anti‐GFAP antibody (Sigma, France, ref C9205, clone G‐A‐5, lot #116M4889V). Microglia were labeled for Iba1 (Ionized Calcium‐binding Adapter Molecule 1) by incubating the slices for 48 h at 4 °C with a rabbit monoclonal anti‐Iba1 antibody (Abcam, France, ref 178846, clone [EPR16588], lot #GR3185035‐1), rinsed 3 times, then incubated for 24–hours at 4°C with a secondary anti‐rabbit IgG (H + L) Alexa‐488 antibody (Life Technologies, France, ref A21206, lot #1927937). All antibodies were diluted 1:1000 in PBS‐Triton‐BSA 0.2%. Cell nuclei were labelled with bis‐benzimide (0.5 µg mL^−1^, Hoechst 33258, Sigma, France). Preparations were mounted in fluoroshield mounting medium (Sigma, France). IHC images from brain stem sections were acquired on a high capacity digital slide fluorescence scanner (Pannoramic SCAN P150, 3D HISTECH) at 20x resolution, using the extended focus function scanner to generate images with extended depth of field from acquired Z‐stacks (11 images, 1 µm interval, 10 µm total thickness).

Iba1‐labeled microglial cells within the AP were manually counted on 1 slice per mouse within the AP and within the left and right NTS delineated between the AP, the NTS, and the motor nuclei of the vagus nerve. In the dorsal vagal complex (DVC) sections, the deployment of the astroglial barrier was quantified by measuring i) the total GFAP area delineating the border between the AP and the NTS on images converted in binary format using an automatic thresholding method (Threshold: “Li” algorithm) on Image J software as previously described,^[^ ^93^ ^]^ and ii) 5 widths representative of the thickness of the barrier: dorsal‐left, ventral left, dorsal right, ventral right, ventral central. Measurements were performed on 1 slice per mouse, each slice being identically positioned to allow comparisons between groups. All measurements were performed by the same experimenter who was blinded to the group assignments.

### Striatum, Hypothalamus and Area Postrema Gene Expression

Hypothalamus and AP total RNA were extracted using miRVANA RNA isolation kit (Ambion, Austin, TX, USA), following manufacturer's instructions as described previously.^[^ ^92^ ^]^ Reverse transcription was performed from 1.8µg of total RNA from the hypothalamus and striatum and 1µg of total RNA from the AP using the cDNA high capacity reverse transcription kit (Applied Biosystems, Wattham, Massachusetts, USA) following manufacturer's instructions. In the striatum, cDNAs diluted 1/7 were amplified by real time PCR using commercially available FAM‐labeled Taqman probes (ThermoFisher Scientific, France), [Opioid Receptor Mu 1 (*oprm1*, Mm01188089 ml), Proenkephalin (*penk*, Mm01212875_m1), Tyrosine Hydroxylase (*th*, Mm00447557_m1), dopamine receptor D1 (*drd1*, Mm02620146_s1), dopamine receptor D2 (*drd2*, Mm00438545_m1), Solute Carrier Family 6 Member 3 (*slc6a3*, Mm00438388_m1)]. Ribosomal protein lateral stalk subunit P0 (*rplp0*, Mm00725448_s1) and β‐actin (*actb*, Mm02619580_g1) were used as reference genes.

In the hypothalamus and AP, gene expression of a total of 48 known candidate genes was studied and analyzed using real time TaqMan Low‐Density Array (TLDA). Pre‐designed 384‐well format gene expression assays were used for the experiments (ThermoFisher Scientific, France). The primers and probe for each assay were preloaded onto the designated well. Forty‐three candidate genes involved in regulation of food intake, brain plasticity, blood‐brain barrier, and microglia, astrocytes & inflammation were selected. In addition, five genes, *actb*, *rplp0*, glyceraldehyde‐3‐phosphate dehydrogenase (*gapdh*), phosphoglycerate kinase 1 (*pgk1*), and 18S ribosomal RNA (*18s*), were selected for spotting on the TLDA cards as endogenous controls. The genes and their functional relevance are listed in Table S1 (Supporting Information).

For both, real time PCR and TLDA, the relative expression levels of target genes were calculated using the 2‐ΔΔCT method considering the geometric mean of reference genes expression levels for determining the ΔCT for each sample and the CTRL as the reference group to determine the ΔΔCT.

### Statistics

Data were not transformed before statistical analysis and are presented as means ± SEM. Sample sizes ranged between 5 and 8 mice per group and are indicated in the figure legends.

Means between groups were compared using one‐way and two‐way ANOVA followed by a Tukey post‐hoc test if appropriate for parametric data (body weight gain and food intake in experiment 1), and Mann‐Whitney test for non‐parametric data (experiments 2 & 3) on Prism GraphPad v.7.00 (GraphPad Software, San Diego, USA). Significance was set for P‐value < 0.05.

Microbiota analysis were performed in R (version 4.3.3). Alpha diversity index (Observed species and Shannon index) were analyzed using Mann‐Whitney tests. A permutational multivariate analysis of variance (PERMANOVA) test was performed on the Bray–Curtis and Jaccard matrices using 9999 random permutations and at a significance level of 0.05. Phylum, family, and genus relative abundances were compared using a Mann‐Whitney test. The normalized relative abundance of the functional pathways levels 2 and 3 identified by PICRUSt2 were analyzed using Mann‐Whitney test.

Microbiota sequences are available at https://doi.org/10.57745/OK3ZHQ and all other data are available upon reasonable request to the corresponding authors.

### Ethics Approval

All the animal experiments comply with the ARRIVE guidelines and have been carried out in accordance with the EU Directive 2010/63/EU for animal experiments and have received written agreement from appropriate ethics committees.

## Conflict of Interest

The authors declare no conflict of interest.

## Author Contributions

M.F. and A.S. were the co‐first authors. V.D. and G.B. were the co‐last authors. Conceptualization was performed by G.B., V.D.; Methodology was performed by M.F., G.B., and V.D.; Software was developed by M.M.; Validation was performed by G.B. and V.D.; Formal analysis was performed by M.F., G.B., V.D., and M.M.; Investigation performed M.F., A.S., S.R., M.M., G.C.P., M.M., G.R., M.S.H., L.L.G., C.P., I.D., V.D., and G.B.; Resources acquired by M.F., S.R., G.C.P., L.L.G., I.D., V.D., and G.B.; Data curation performed M.F. and M.M.; the original draft was written by M.F., A.S., I.D., V.D., and G.B.; the review was written and edited by M.F., A.S., I.D., S.B.F., A.B., V.D., and G.B.; Visualization was performed by A.S., I.D., V.D., and G.B.; Supervision by V.D. and G.B.; Project administration performed V.D. and G.B.; Funding acquisition by A.B., V.D., and G.B.

## Supporting information



Supporting Information

Supplemental Table 1

## Data Availability

The data that support the findings of this study are openly available in https://entrepot.recherche.data.gouv.fr/ at https://doi.org/10.57745/OK3ZHQ, reference number 1.
