# Supplementary material for: Weight Cycling Deregulates Eating Behavior in Mice via the Induction of Durable Gut Dysbiosis
Source: Adv Sci (Weinh). 2025 Jun 26;12(32):e01214. doi: 10.1002/advs.202501214 (PMC12407282; doi:10.1002/advs.202501214)
Supplement: Supplementary file 1 — Supporting Information [file ADVS-12-e01214-s002.pptx]

## Slide 1
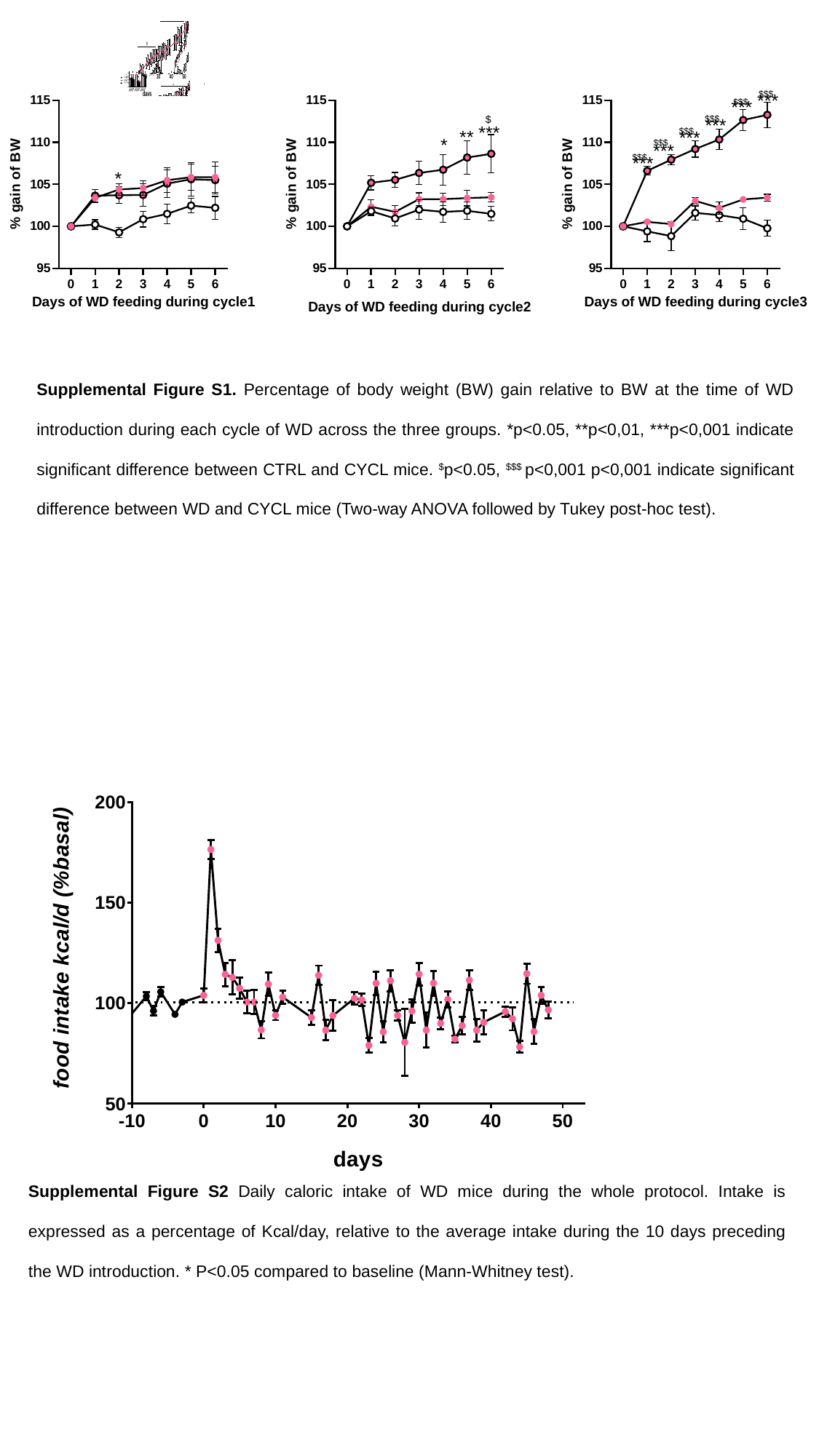

$$$
***
$$$
***
$
$$$
***
***
$$$
**
***
*
$$$
***
$$$
***
*
Supplemental Figure S1. Percentage of body weight (BW) gain relative to BW at the time of WD introduction during each cycle of WD across the three groups. *p<0.05, **p<0,01, ***p<0,001 indicate significant difference between CTRL and CYCL mice. $p<0.05, $$$ p<0,001 p<0,001 indicate significant difference between WD and CYCL mice (Two-way ANOVA followed by Tukey post-hoc test).
Supplemental Figure S2 Daily caloric intake of WD mice during the whole protocol. Intake is expressed as a percentage of Kcal/day, relative to the average intake during the 10 days preceding the WD introduction. * P<0.05 compared to baseline (Mann-Whitney test).

## Slide 2
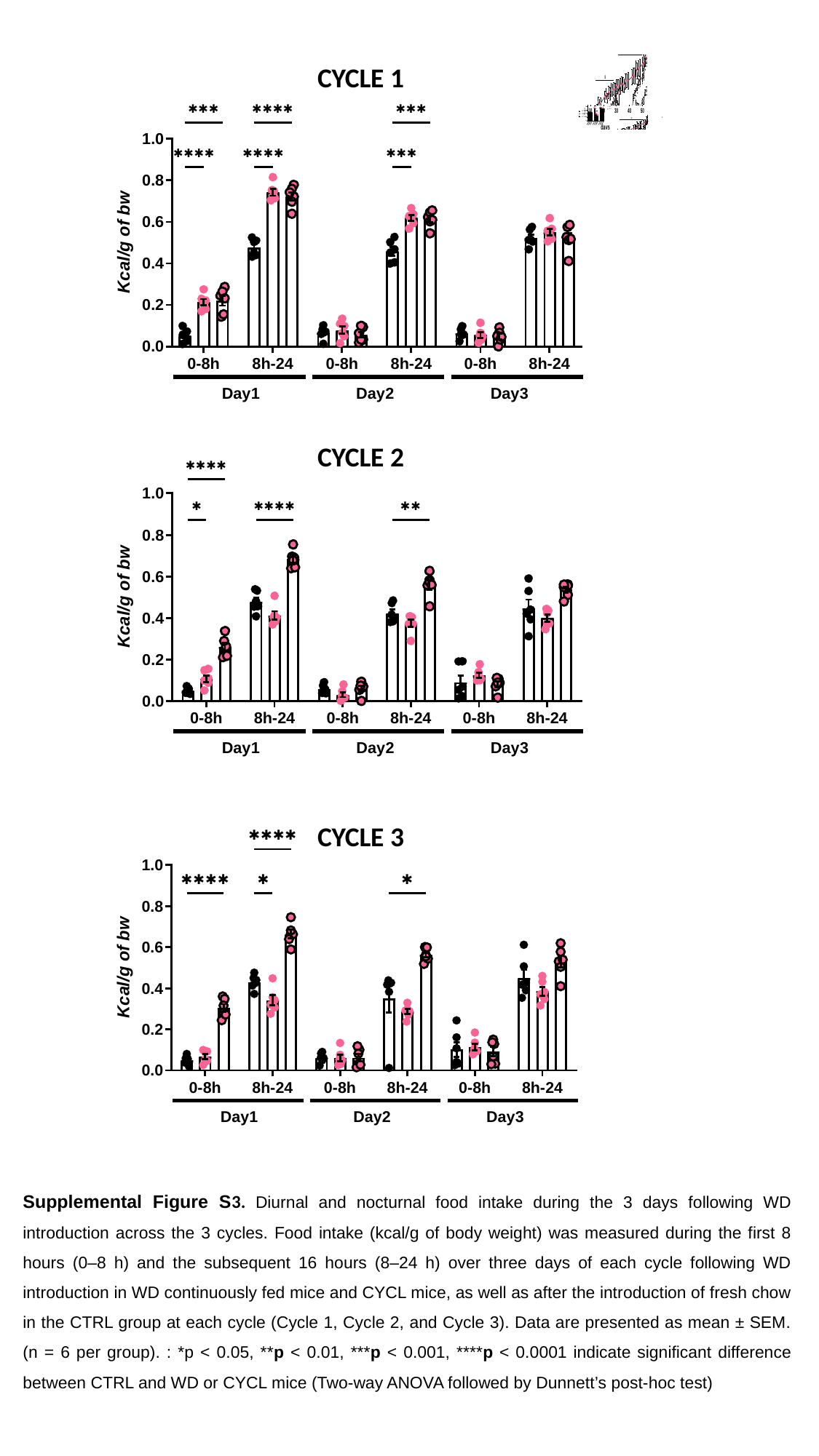

CYCLE 1
CYCLE 2
CYCLE 3
Supplemental Figure S3. Diurnal and nocturnal food intake during the 3 days following WD introduction across the 3 cycles. Food intake (kcal/g of body weight) was measured during the first 8 hours (0–8 h) and the subsequent 16 hours (8–24 h) over three days of each cycle following WD introduction in WD continuously fed mice and CYCL mice, as well as after the introduction of fresh chow in the CTRL group at each cycle (Cycle 1, Cycle 2, and Cycle 3). Data are presented as mean ± SEM. (n = 6 per group). : *p < 0.05, **p < 0.01, ***p < 0.001, ****p < 0.0001 indicate significant difference between CTRL and WD or CYCL mice (Two-way ANOVA followed by Dunnett’s post-hoc test)

## Slide 3
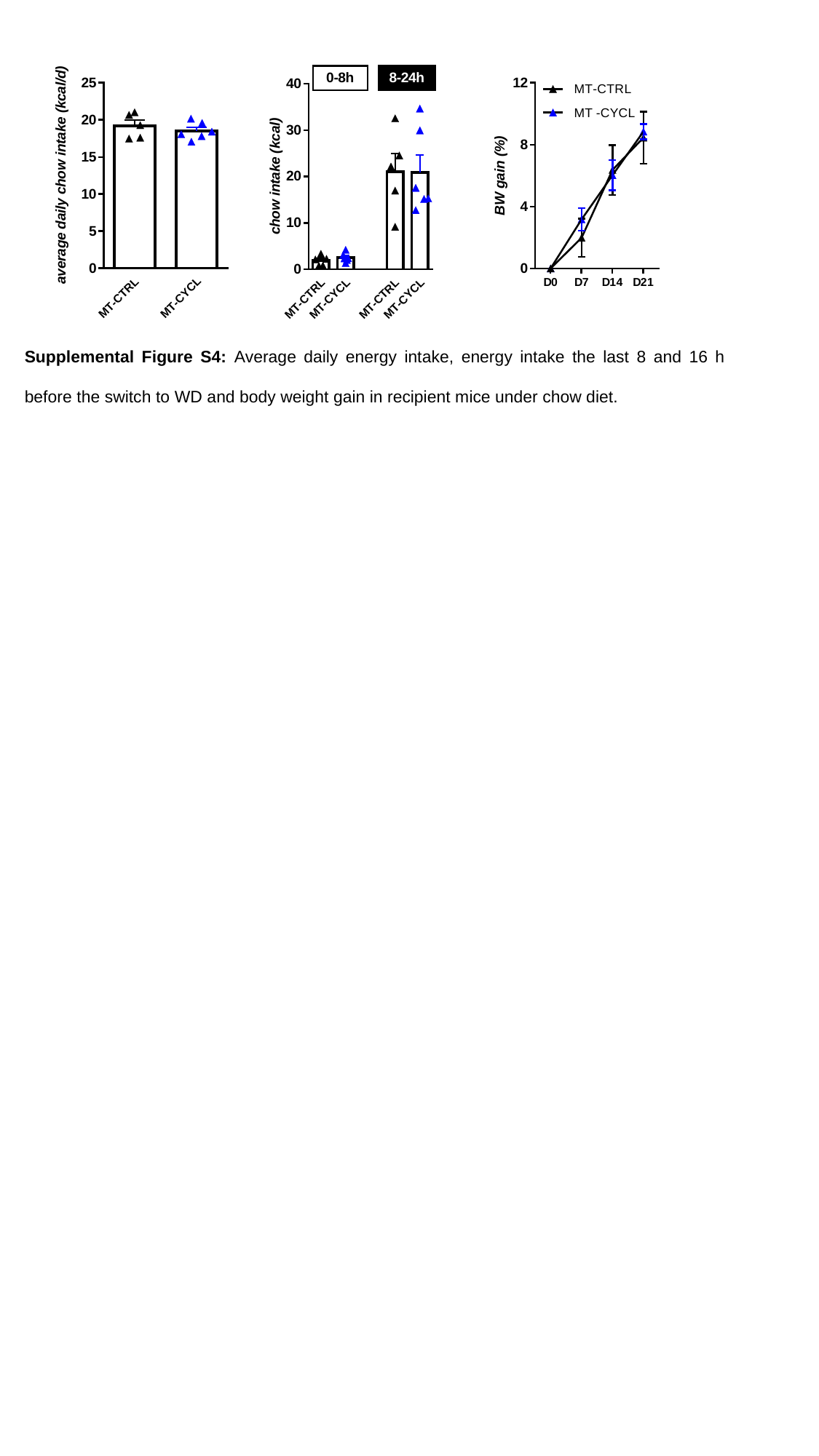

Supplemental Figure S4: Average daily energy intake, energy intake the last 8 and 16 h before the switch to WD and body weight gain in recipient mice under chow diet.
